# Does the use of biological traits predict a smooth landscape of ecosystem functioning?

**DOI:** 10.1002/ece3.6696

**Published:** 2020-08-20

**Authors:** Johanna Gammal, Judi Hewitt, Joanna Norkko, Alf Norkko, Simon Thrush

**Affiliations:** ^1^ Tvärminne Zoological Station University of Helsinki Hangö Finland; ^2^ National Institute of Water and Atmospheric Research Hamilton New Zealand; ^3^ Department of Statistics University of Auckland Auckland New Zealand; ^4^ Baltic Sea Centre Stockholm University Stockholm Sweden; ^5^ Institute of Marine Science University of Auckland Auckland New Zealand

**Keywords:** benthic macrofauna, biodiversity–ecosystem functioning, functional traits, key species, redundancy, spatial patterns

## Abstract

The biodiversity crisis has increased interest in understanding the role of biodiversity for ecosystem functioning. Functional traits are often used to infer ecosystem functions to increase our understanding of these relationships over larger spatial scales. The links between specific traits and ecosystem functioning are, however, not always well established. We investigated how the choice of analyzing either individual species, selected modalities, or trait combinations affected the spatial patterns observed on a sandflat and how this was related to the natural variability in ecosystem functioning. A large dataset of 400 benthic macrofauna samples was used to explore distribution patterns. We hypothesized that (1) if multiple species (redundancy) represent a trait combination or a modality their spatial patterns would be smoothed out, and (2) the lost spatial variability within a trait combination or modality, due to the smoothing effect, would potentially affect their utility for predicting ecosystem functioning (tested on a dataset of 24 samples). We predicted that species would show heterogeneous small spatial patterns, while modalities and trait combinations would show larger and more homogeneous patterns because they would represent a collection of many distributions. If modalities and trait combinations are better predictors of ecosystem functioning than species, then the smoother spatial patterns of modalities and trait combinations would result in a more homogeneous landscape of ecosystem function and the number of species exhibiting specific traits would provide functional redundancy. Our results showed some smoothing of spatial patterns progressing from species through modalities to trait combinations, but generally spatial patterns reflected a few dominant key species. Moreover, some individual modalities and species explained more or equal proportions of the variance in the ecosystem functioning than the combined traits. The findings thus suggest that only some spatial variability is lost when species are combined into modalities and trait combinations and that a homogeneous landscape of ecosystem function is not likely.

## Graphical abstract

Functional traits are often used to infer ecosystem functioning across larger scales, but how do they reflect species distributions and how well do they actually explain the natural spatial variability in ecosystem functioning? This was examined with a rarely seen extensive dataset of benthic macrofauna samples and solute flux measurements across the sediment–water interface.

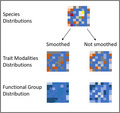

## 1 INTRODUCTION

Environmental change and biodiversity loss have increased interest in the role of biodiversity for ecosystem functioning. The overall goal of biodiversity–ecosystem function (BEF) research has been to understand why biodiversity matters and how ecosystems may be able to maintain the functions that support ecosystem services despite environmental degradation (Srivastava & Vellend, 2005). Evidence of positive relationships between biodiversity and functioning is piling up, but the patterns are often context‐dependent and equivocal (Gamfeldt et al., 2015; Reiss, Bridle, Montoya, & Woodward, 2009). The question has, however, moved on from whether biodiversity has an effect, to how BEF relationships change in space, time, or under specific environmental conditions. There has also been a demand for studies on larger scales in order to support ecosystem‐based management decisions (Gamfeldt et al., 2015; Reiss et al., 2009; Snelgrove, Thrush, Wall, & Norkko, 2014). In order to enhance the understanding of BEF relationships, studies have not only started to consider multiple functions (Byrnes et al., 2014; Gagic et al., 2015; Hiddink, Davies, Perkins, Machairopoulou, & Neill, 2009; Villnäs et al., 2013), but, more importantly, the scope of biodiversity components analyzed has been widened (Purvis & Hector, 2000; Reiss et al., 2009; Thrush et al., 2017).

The use of functional traits—defined as any morphological, physiological, phenological, or behavioral feature that can be measured on the level of an individual and can be used to describe its performance (Violle et al., 2007)—has advanced our understanding of BEF relationships. While species richness as a biodiversity metric assumes that all species are equal with respect to function, functional traits demonstrate that species differ in their roles in ecosystem functioning (Bengtsson, 1998; Walker, 1992). If many species in a community express the same traits (redundancy), they might be complementary and support continued function even if a species is lost (i.e., the insurance hypothesis, Yachi & Loreau, 1999). Functional redundancy is thus an important aspect of resilience of ecosystems (e.g., Walker, 1992).

Redundancy in a functional group may also depend on the number and specificity of the traits that are used to form the group, as this will define the number of species that contribute (Micheli & Halpern, 2005). Thus, spatial scales of heterogeneity in the distribution of species with functionally similar traits become important. The variability in species abundance and occurrence within a functional group can be high in heterogeneous landscapes (Hewitt, Thrush, & Dayton, 2008; Walker, 1992; Wellnitz & Poff, 2001), with redundancy within functional groups affected by the spatial variation in species composition (Naeem, Duffy, & Zavaleta, 2012). Additionally, a functional group containing low species richness and low abundance would not necessarily be considered to contain redundancy, but if the group occurs widely over a landscape, it might still be important for the system's resilience (Greenfield, Kraan, Pilditch, & Thrush, 2016).

Scale is thus important in all aspects of biodiversity–ecosystem function relationships. The relationship between biodiversity and ecosystem functioning depends on the investigated temporal (Cardinale et al., 2007; Stachowicz, Graham, Bracken, & Szoboszlai, 2008; Tilman et al., 2001) and spatial scales of heterogeneity (Cardinale et al., 2011; Dimitrakopoulos & Schmid, 2004; Dyson et al., 2007; Godbold, Bulling, & Solan, 2011; Griffin et al., 2009; Raffaelli, 2006). Such strong scale‐dependence makes it challenging to validate how well functional traits work as surrogates for functioning, and how the heterogeneity of landscapes is reflected in terms of functional traits.

Estuarine and coastal marine benthic ecosystems are very suitable for BEF studies due to their high biodiversity, encompassing trophic levels, ease of sampling, and the naturally occurring environmental gradients and diverse set of habitats within a relatively restricted distance (e.g., Snelgrove et al., 2014). We used a large dataset of 400 benthic macrofauna samples from an extensive sandflat to explore spatial patterns of species distributions, trait modalities, and trait combinations. From a smaller dataset of 24 samples in a subset of locations, we investigated the ability of individual species, trait modalities, and trait combinations to explain the ecosystem multi‐functionality related to nutrient recycling. In this study, the investigated trait combinations were selected based on their importance for nutrient recycling processes at the sediment–water interface (Kristensen et al., 2012; Norkko, Villnäs, Norkko, Valanko, & Pilditch, 2013; Solan et al., 2004), which were investigated on the sandflat through solute flux measurements (Thrush et al., 2017). More specifically, we investigated how the choice of analyzing either individual species, selected modalities, or trait combinations affected the spatial patterns observed on the sandflat and how this was related to the natural variability in ecosystem functioning.

**FIGURE 1:**
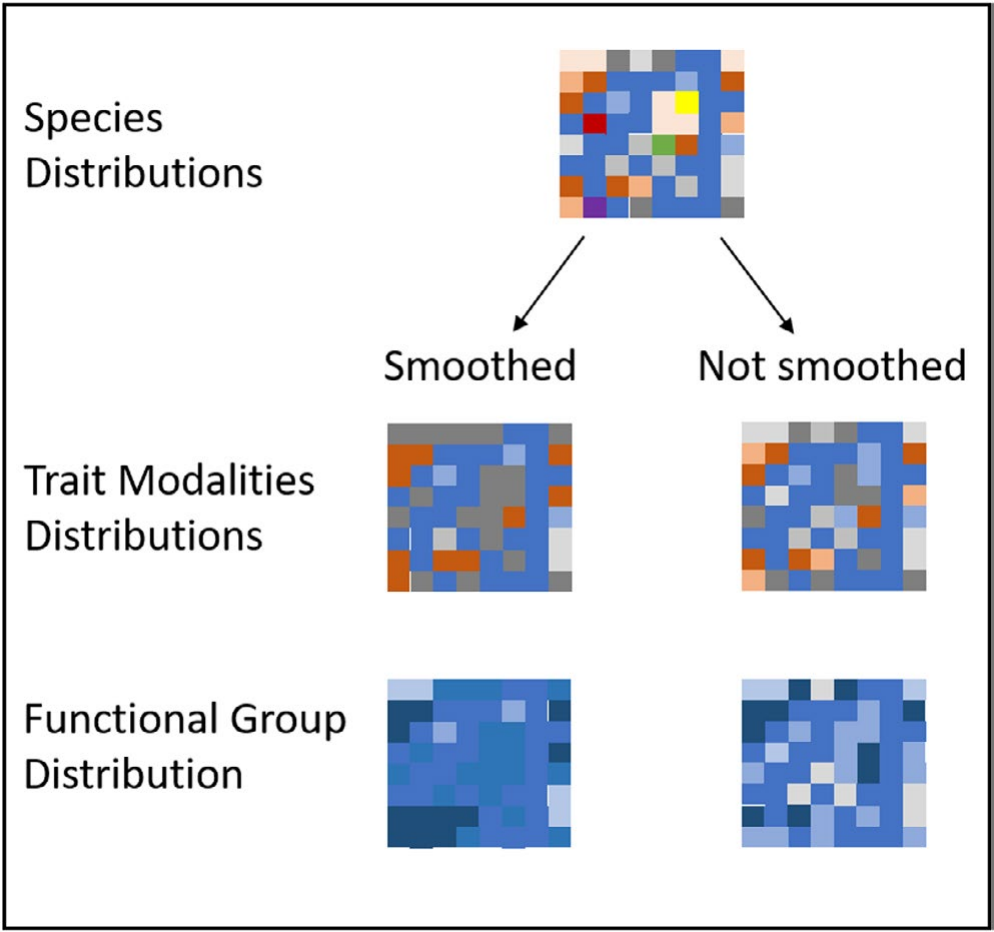
Schematic illustration of expected spatial patterns in case of smoothing or no smoothing effects. Each colour depicts a species, modality, or functional group respective to each level, whereas the shades illustrate the abundance of each species, modality, and functional group at each level. A smoothing effect would be indicated by larger homogeneous patches at the modality level and subsequently lead to a homogeneous distribution of the functional group. Whereas, the nonsmoothed patterns would stay more heterogeneous with smaller patches and variable abundances on the modality level, and the distribution of the functional group would be more variable across the seafloor

We hypothesized that (1) if multiple species represent a trait combination or a modality, their individual spatial patterns would be smoothed over. That is, the species would show heterogeneous small spatial patterns across the sampled area, while the modalities and the trait combinations would show larger and more homogeneous patterns because they would represent a collection of many partially overlapping distributions (Figure 1). We further hypothesized (2) that this lost spatial variability within a trait combination or modality would not affect their ability to predict ecosystem functioning, that is, they would be better predictors than individual species. If both hypotheses were found to be true, then the smoother spatial patterns of the modalities and trait combinations would result in a smoother landscape of ecosystem function and the number of species exhibiting traits would provide functional redundancy.

## 2 MATERIAL AND METHODS

### 2.1 Study area and sampling

The sampling was conducted in Kaipara Harbour (36°39ʹS, 174°29ʹE), New Zealand. An extensive sandflat (300,000 m^2^) was surveyed from the high‐ to low‐water mark in April 2012. A total of 400 macrofaunal cores (13 cm diameter, 20 cm deep) were sampled on a grid (1,000 × 300 m) of four transects with a repeated sequence of sampling intervals (0.3, 1, 5, 10, 20, and 50 m) along each transect, enabling identification of spatial patterns at multiple spatial scales (e.g., Greenfield et al., 2016; Kraan, Dormann, Greenfield, & Thrush, 2015). The macrofaunal cores were sieved (500 µm mesh) and the residue preserved in 70% isopropyl alcohol. The sampled area included a substantial variation in benthic macrofauna communities, sediment mud content, and cover of seagrass (Zostera muelleri, Kraan et al., 2015).

From the extensive survey data, 24 experimental locations with varying abundance and richness of macrofauna species with functional traits likely to affect nutrient processes in the sediments were selected. These locations correspond to the control plots in the experiments described in Thrush et al. (2017) and shown in their Appendix S1: Figure S1. Solute fluxes (oxygen, ammonium, and phosphate) across the sediment–water interface were measured at each location in March 2014 through chamber incubations and a multivariate response matrix of the normalized solute fluxes were used as a measure of ecosystem multifunction. At the same time, the macrobenthic community was sampled after the flux measurements had been made, using 2 replicate cores (13 cm diameter, 15 cm deep) at each location. The macrofauna cores were sieved (500 µm mesh) and preserved in 50%–70% isopropyl alcohol, before being identified to the lowest taxonomic level possible and counted.

### 2.2 Flux measurements

Chamber bases (50 × 50 × 10 cm height) were pressed approximately 5 cm down into the sediment during low tide, and when the water level during incoming tide reached approximately 30 cm, the chambers were sealed with Perspex domes. Opaque shade cloths were used to ensure darkness within the chambers throughout the incubation period, which occurred during a midday high tide of approximately 4 hr. Dark incubations were used to control for photosynthetic activities and nutrient uptake by microphytobenthos and seagrass. To measure solute concentrations, water samples (60 ml) from the chambers were collected through sampling ports at the beginning and end of the incubations. The oxygen concentrations were measured with an optical probe, whereas the nutrient samples were filtered through a 0.8‐µm glass fiber filter and stored frozen until analysis. The solute fluxes were calculated as (*C*_end_–*C*_initial_) × *V*/*A* × *T*, where *C* is solute concentration (µM/L), *V* is the incubated sea water volume (*L*), *A* is the area (m^2^) incubated, and *T* is the incubation time (h). The inorganic nutrient concentrations (PO_4_^3−^ and NH_4_^+^) in the water samples from the flux chambers were analyzed colorimetrically with a Lachat QuickChem 8000 auto analyzer.

### 2.3 Trait data

We focused on functional traits that are known to have an impact on nutrient cycling and fluxes at the sediment–water interface by using traits related to vertical movement of sediment particles and solutes, creation of sediment topographic features, body size, and degree of motility (Norkko et al., 2013, 2015; Solan et al., 2004; Villnäs et al., 2013). These traits were expected to affect solute fluxes in the sediment by moving sediment particles and organic material, pumping pore water, and changing sediment topography (Thrush et al., 2017; Volkenborn et al., 2012; Woodin et al., 2016). Where species exhibited attributes of several modalities within one trait, fuzzy probabilities were used to assign species to modalities (Chevenet, Dolédec, & Chessel, 1994), with allocation across traits summing to 1. First, four trait combinations were created from a fuzzy coded modality by species matrix. Three different ways of producing a vertical mixing combination trait were explored and one for surface modification (Appendix S1). The trait combination *mixing* contained the sum of the modalities *surface to depth* and *depth to surface*, describing the direction of particle and solute movement. The trait mixing*L contained the same modalities but only the *large* taxa (species > 20 mm), and the trait mixing*size*motility contained the same modalities but weighted by *size* (1 = small <5 mm, 2 = medium 5–20 mm, 3 = large >20 mm) and *motility* (1 = sedentary/movement in a fixed tube, 2 = limited movement, 3 = freely motile). Body size, in addition to direction of particle movement, was included since size has been shown to play an important role for nutrient dynamics at the sediment–water interface (e.g., Norkko et al., 2013; Sandnes, Forbes, Hansen, Sandnes, & Rygg, 2000; Thrush, Hewitt, Gibbs, Lundquist, & Norkko, 2006). Larger organisms are for example likely to move more sediment and water, thus creating stronger gradients in pore water pressures within sediments (Volkenborn et al., 2012). The trait *surface modification*, describing fauna‐produced structures in or on the sediment, contained the modalities *permanent burrow*, *tube structure*, *simple hole or pit*, *mound,* and *trough*. After every species was assigned a trait value, the values were abundance weighted and a sum across species was calculated to result in a single value for each trait combination and included modality in each sample (see Appendix S2 for species allocations). The abundance weighted traits were calculated in the same way for both datasets.

### 2.4 Statistical analysis

#### 2.4.1 Trait combination selection

Initially, Distance based linear modeling (DistLM; PERMANOVA + add on in PRIMER v7, Anderson, Gorley, & Clarke, 2008) was used to identify which of the four trait combinations could best account for the variation in the multifunctioning. A multivariate response matrix of the normalized solute fluxes of oxygen, ammonium, and phosphate was used as the measure of multifunctioning because solute fluxes are very variable along environmental gradients and variably affected by environmental and biological factors. Thus, combining them facilitates detection of robust BEF relationships even across heterogeneous landscapes (e.g., Gammal, Järnström, Bernard, Norkko, & Norkko, 2019; Link, Chaillou, Forest, Piepenburg, & Archambault, 2013; Villnäs et al., 2013). DistLM was conducted on the small dataset (24 locations), with stepwise selection procedure and an AIC_c_ stopping criterion. To select the trait combinations that best explained multifunctioning, we used the results of both the marginal tests—indicating the proportion of the variance in the multivariate response matrix each predictor accounts for when fitted one at a time; and sequential tests—indicating which combination of predictors accounts for the largest proportion of the variance.

Hypothesis 1 Individual species spatial patterns are smoothed over as they are combined in modalities and finally trait combinations.

The two trait combinations (mixing*L and *surface modification*) that best explained multifunction (Table 1) were further investigated across the survey data set (400 locations). The spatial distribution patterns of the traits, and the modalities and species which formed them were explored using spatial autocorrelograms of Moran's I coefficients in the program Spatial Analysis in Macroecology (SAM; Rangel, Diniz‐Filho, & Bini, 2010). Moran's I coefficients identify the degree of correlation between samples with increasing distance from each other (Dormann et al., 2007; Legendre & Fortin, 1989), and values range from +1 (strong spatial correlation), 0 (no correlation), to −1 (negative correlation). The analyses were run with equal distance in each distance class, that is, average correlations between samples laying within 0–20 m of each other, 20–40 m of each other, and so on were determined. Before each correlogram of the Moran's I coefficients were explored, a global test was performed to account for the multiple tests conducted. The global tests were performed through checking that each correlogram contained at least one value that was significant at a level of *a*ʹ = *a*/*v*, where *a* was 0.05 and *v* was the number of tests performed (Oden, 1984).

**TABLE 1:** DistLM marginal test with the investigated trait combinations as predictors of the multifunction (the solute fluxes PO_4_^3‐^, NH_4_^+,^ and O_2_)

Variable	SS(trace)	Pseudo‐*F*	*p*	Proportion explained
Mixing	6.6	2.3	.0834	0.095
Mixing*L	**16.1**	**6.7**	**.0002**	**0.233**
Mixing*size*mobility	7.5	2.7	.0567	0.108
Surface modification	**8.5**	**3.1**	**.0328**	**0.123**

Note

The significant trait combinations sediment mixing by large taxa (Mixing*L) and *Surface modification* are in bold.

Autocorrelograms provide information on average spatial patterns and do not necessarily indicate similar specific spatial location (Sokal & Oden, 1978). Therefore, to further investigate the patch locations of the modalities and species included in the two investigated trait combinations, Spearman correlations were used to compare the dissimilarity matrices (co‐occurrences) of the variables across the sandflat. High positive Spearman rho coefficients of two variables exhibiting spatial patchiness suggest that the spatial location of the patches coincide, high negative coefficients suggest avoidance, and low coefficients suggest that there is no relationship. The results thus focus on the strength of the correlations, and due to the number of correlations conducted on nonindependent variables, *p*‐values are not reported. Supporting maps were produced in the program SAM to illustrate the distribution of the trait combinations, modalities, and the abundance of species across the sandflat.

Hypothesis 2 Multifunctioning is better predicted by trait combinations than by modalities or individual species.

DistLM was run between the normalized solute flux matrix and the two trait combinations, as described above. However, this time, all modalities and species contributing to the trait combinations were included as predictors.

## 3 RESULTS

### 3.1 Trait selection

The DistLM marginal tests revealed that the trait combination mixing*L (including the modalities mixing from *surface to depth* and *depth to surface* by the *large* species) and the trait combination *surface modification* (including the modalities *permanent burrow*, *tube structure*, *simple hole or pit*, *mound*, and *trough*) individually were significant and accounted for the largest proportions of the variance explained in the multifunction solute fluxes (Table 1). The mixing*L trait combination accounted for 23%, and the *surface modification* trait combination accounted for 12% of the variance in the multifunction. The two additional trait combinations that were initially explored, *mixing* (including modalities mixing *surface to depth* and *depth to surface*) and *mixing depending on size and motility* (including modalities mixing *surface to depth* and *depth to surface* weighted by *size* and *motility*), were not significant in the marginal tests, and therefore not further explored.

A total of 9 species contributed to the trait combination mixing*L across the whole sandflat, whereas 70 species contributed to the trait combination *surface modification* (Appendix S2). The two modalities in mixing*L, *surface to depth* and *depth to surface* had 7 and 8 species, respectively, with 6 species in common. The most abundant species within this trait combination were the bivalves *Austrovenus stutchburyi* and *Macomona liliana*. In order to constrain the number of further analyses of individual species, species with less than 10 individuals across all 400 samples were referred to a group called “others.” Within the trait combination mixing*L 3 species were combined to such a group. The 5 modalities within *surface modification* encompassed a variable number of species, 8–26 species. The most abundant species in this trait combination were the bivalves *M. liliana* (trough), *Paphies australis,* and *Soletellina siliqua* (simple hole or pit). The group “others” contained 26 species.

### 3.2 Hypothesis 1—spatial patterns of the trait combinations, modalities, and species (autocorrelation)

**FIGURE 2:**
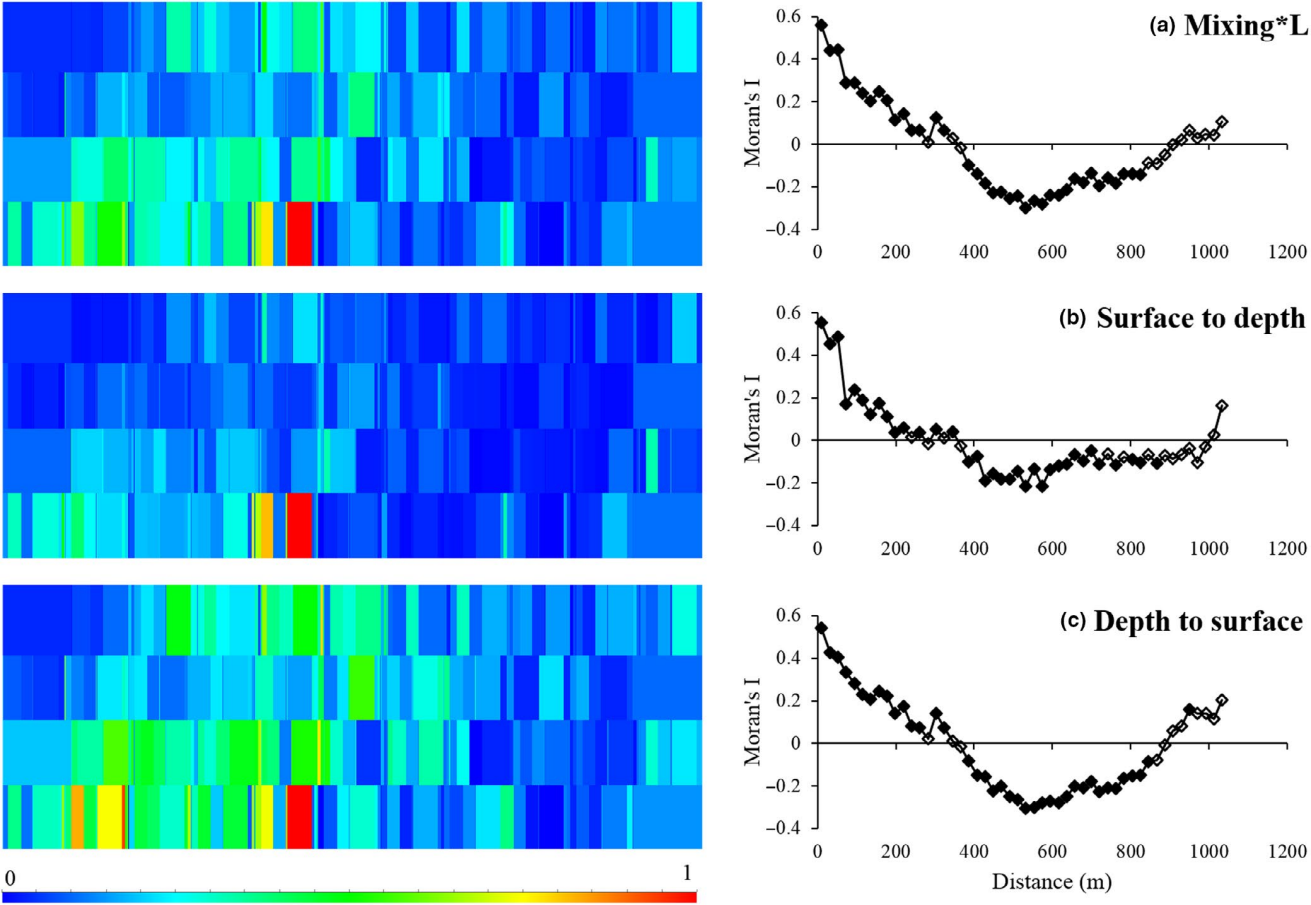
Maps of the spatial distribution (on the left) and correlograms (on the right) of (a) the trait combination mixing*L, (b) the modality surface to depth and (c) the modality depth to surface. The data in the maps are normalized to run from 0 to 1, to illustrate the patch patterns and locations of the patches across the sandflat (1 km × 0.3 km). The maximum values of the raw data in each map were (a) 31.3, (b) 13.9, and (c) 17.4. Filled symbols in the correlograms indicate significant values

The correlograms of the Moran's I coefficients indicated that the two investigated trait combinations, the modalities and species, showed distribution patterns at many different scales. Mixing*L had a large‐patch pattern (300 m), as did one of its modalities *depth to surface* (300 m), indicated by significant positive values at the first distance classes followed by negative values but increasing values toward the last distance classes (Figure 2; Appendix S3). The modality *surface to depth* had multiple patch patterns (50 m patches within 200 m patches), indicated by an oscillation of significant positive coefficient values followed by negative values. The species in these modalities exhibited patterns at many scales (Appendices S3 and S4). The species included in the trait combination mixing*L showed patterns of; patches of 300 m (1 species); patches < 100 m (2 species); patch on gradient (2 species); and multiple patches (2 species).

**FIGURE 3:**
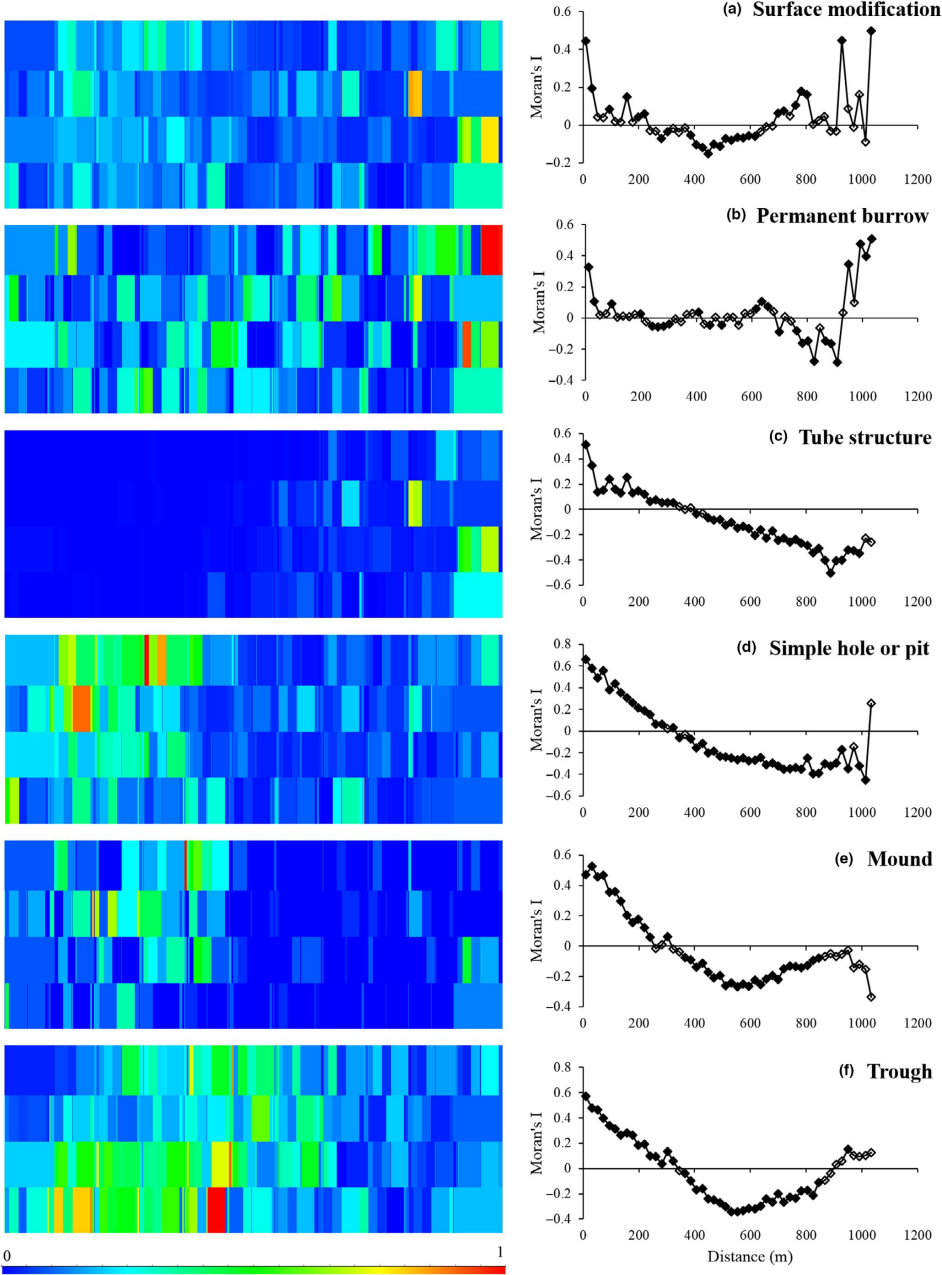
Maps of the spatial distribution (on the left) and correlograms (on the right) of (a) the trait combination surface modification, the modalities (b) permanent burrow, (c) tube structure, (d) simple hole or pit, (e) mound, and (f) trough. The data in the maps are normalized to run from 0 to 1, to illustrate the patch patterns and locations of the patches across the sandflat (1 km × 0.3 km). The maximum values of the raw data in each map were (a) 211, (b) 21, (c) 192, (d) 75, (e) 12, and (f) 32. Filled symbols in the correlograms indicate significant values

The trait combination *surface modification* appeared to be distributed in 30–90 m patches within 300–400 m patches (Figure 3; Appendix S3). Its modalities exhibited many different patterns. The modality *permanent burrow* was distributed in small patches (30–90 m); *tube structure* showed small patch pattern (30–50 m) on a gradient; *simple hole or pit* showed large‐patch pattern (300–350 m); while *mound* and *trough* were distributed in patches of 200 m and 300 m, respectively. The species included in the trait combination *surface modification* showed patterns of; patches 100–300 m (4 species); patches < 100 m (11 species); gradients with or without patches (14 species); multiple patches (8 species); and random distributions (8 species; Appendices S3 and S4).

### 3.3 Hypothesis 1—spatial correlations of trait combinations, modalities, and species abundances

Spearman correlations were used to investigate the spatial location of the patches in relation to each other across the sandflat. The modalities *surface to depth* and *depth to surface*, contributing to the trait mixing*L, were highly correlated to the trait combination (i.e., Spearman rho 0.74 and 0.95, respectively; Table 2). Furthermore, the modalities also exhibited a correlation of > 0.5 (0.59), indicating these patches overlapped even if they showed slightly different spatial patterns (see also Figure 2). Thus, these modalities contributed to the trait combination mixing*L in an additive manner.

**TABLE 2:** Spearman correlations between the distributions of the trait combination mixing*L and the included modalities and species abundances

	Mixing*L	Surface to depth	Depth to surface	*Austrovenus stutchburyi*	*Macomona liliana*	*Hemiplax hirtipes*	*Platynereis australis*	*Glycera americana*	*Cyclomactra ovata*
Surface to depth	**0.74**								
Depth to surface	**0.95**	**0.59**							
*Austrovenus stutchburyi*	0.35	**0.63**	0.28						
*Macomona liliana*	**0.66**	0.31	**0.77**	0.08					
*Hemiplax hirtipes*	0.03	0.08	0.01	0.07	−0.01				
*Platynereis australis*	−0.05	−0.04	−0.05	−0.04	−0.05	0.27			
*Glycera americana*	−0.01	‐0.00	−0.01	0.00	−0.01	0.13	0.09		
*Cyclomactra ovata*	−0.06	0.08	−0.05	−0.04	−0.05	0.04	0.08	−0.01	
Others	‐0.00	0.05	−0.02	0.04	−0.04	0.07	0.02	0.07	0.04

Note

Bold numbers indicate Spearman's rho ≥ 0.5.

The species distributions were not strongly correlated (all < 0.2; Table 2), indicating that the species were relatively scattered across the sandflat. A couple of abundant species were correlated to the modalities and the trait combination. *Austrovenus stutchburyi* correlated (0.63) with the modality *surface to depth*, while *Macomona liliana* correlated (0.77) with the modality *depth to surface*, which (together with their matching patch pattens and dominating contributions; Appendix S3) indicated that these key species were driving the emerged spatial pattern of the respective modalities. Moreover, *M. liliana* was also correlated (0.66) with the trait mixing*L, which again together with the matching scales of the patches, suggested that this species could be a driver of the distribution of the trait mixing*L.

There were no strong correlations between the modalities contributing to the trait combination *surface modification*, and no modality was strongly correlated with the trait combination (Table 3). The lack of correlations together with a visual inspection of the maps (Figure 3) indicated that the modalities contributed to the trait combination *surface modification* in a complementary manner across the sandflat.

**TABLE 3:** Spearman correlations between the distributions of the trait combination *surface modification* and the included modalities

	Surface modification	Permanent burrow	Tube structure	Simple hole or pit	Mound
Permanent burrow	0.09				
Tube structure	0.14	0.06			
Simple hole or pit	0.40	0.00	0.06		
Mound	0.07	−0.01	0.04	0.11	
Trough	0.12	0.01	0.06	0.02	−0.01

There were no strong correlations among the species distributions belonging to the trait *surface modification* (all < 0.45; Appendix S5), indicating that the species were scattered across the sandflat. No species was directly correlated with the trait *surface modification,* and correlations > 0.5 only occurred between the modality *tube structure* and 4 species (*Euchone* sp., *Owenia petersonae*, *Macroclymenella stewartensis* and *Phoronis* sp.; Appendix S5).

### 3.4 Hypothesis 2—predictions of multifunction by trait, modalities, and species

Marginal and sequential tests in the DistLM analysis were used to investigate how useful trait combinations, modalities, and species were in predicting multifunction, in order to further elucidate if smoothing of the spatial patterns on the trait level would result in decreased ability to predict functionality. The results indicated that some modalities and individual species accounted for a larger proportion of the variance in the multifunction than the actual trait combinations mixing*L and *surface modification* (Table 4). For instance, the abundance of the bivalve *Austrovenus stutchburyi* (26%) could alone explain a larger proportion of the variance in the multifunction than either of the two trait combinations (cf. 23% and 12%). Three of the five modalities included in *surface modification* also explained more variance than the trait combination *surface modification* (see Table 4; *simple hole or pit*, *tube structure*, *trough*).

**TABLE 4:** Results from the marginal and sequential tests in DistLM analyses, marginal tests indicate the proportion of the variance each predictor accounts for in terms of multifunction, that is, the phosphate (PO_4_^3−^), ammonium (NH_4_^+^), and oxygen (O_2_) fluxes (*n* = 24). Sequential tests indicate which combination of predictors within the different levels (trait combination, modalities, and species) best explains the variance of the multifunction

Marginal tests	Pseudo‐*F*	*p*	Proportion explained
Trait combinations			
Mixing*L	6.68	<.001	0.23
Surface modification	3.09	.030	0.12
Modalities			
Surface to depth			
Depth to surface			
Permanent burrow			
Tube structure	6.43	<.001	0.23
Simple hole or pit	5.75	.001	0.21
Mound			
Trough	5.64	.001	0.20
Large	4.64	.007	0.17
Species			
*Austrovenus stutchburyi*	7.78	<.001	0.26
*Paphies australis*	2.74	.049	0.11
*Owenia petersonae*	3.62	.018	0.14
*Pseudopolydora* thin	5.86	.001	0.21
*Macroclymenella stewartensis*	6.16	.001	0.22
*Armandia maculata*	3.49	.022	0.14
*Austrominius modestus*	3.65	.019	0.14
Nemertean sp.	2.74	.049	0.11

Sequential tests	Pseudo‐*F*	*p*	Proportion explained	Cumulative proportion explained	AICc
Trait combinations					
Mixing*L	6.683	.001	0.23	0.23	23.55
Surface modification	4.373	.006	0.13	0.37	21.64
Modalities					
Tube structure	6.435	<.001	0.23	0.23	23.76
Trough	5.685	.005	0.16	0.39	20.64
Species					
*Austrovenus stutchburyi*	7.784	<.001	0.26	0.26	22.65
*Macroclymenella stewartensis*	7.704	<.001	0.20	0.46	17.78
*Magelona dakini*	5.051	.002	0.11	0.57	15.28
*Nicon aestuariensis*	3.666	.021	0.07	0.64	14.27
Hesionidae	3.396	.031	0.06	0.70	13.73

Note

Only significant (*p* < .05) results are presented.

## 4 DISCUSSION

Through exploring spatial distributions of two trait combinations, the included modalities and species, we investigated the potential for functional redundancy to occur over space, and additionally, the degree to which variability in functional traits may predict spatial variability in ecosystem multifunctioning across an extensive sandflat area. We found some evidence for our first hypothesis; modalities and trait combinations would show larger and more homogeneous spatial patterns than individual species because they would represent a collection of many distributions, allowing functional redundancy to occur. However, we did not find clear evidence for our second hypothesis; that this loss of spatial variability would not prevent the trait combinations predicting ecosystem functioning better than individual species.

We chose to investigate two trait combinations that have been proven to be important for nutrient recycling processes (measured as solute fluxes across the sediment–water interface). One trait combination described the potential for vertical movement of particles and solutes by large macrofauna species (mixing*L), and the other trait combination described structures the fauna creates at the sediment surface (*surface modification*). The species distributions were not strongly correlated with each other, and thus, the potential for functional redundancy to occur over space was found in each modality and in each trait combination. The modalities described the direction of the vertical movement of particles and solutes and the specific structures the species creates. The modalities thus describe different attributes of the included species, but they all describe an effect on the surrounding conditions, which consequently has an effect on the biogeochemical processes and thus the nutrient recycling within the sediment. The results further indicated that species may have varying spatial distribution patterns and that this spatial variability was smoothed out on the level of modalities and further smoothed out on the trait combination level. However, some individual modalities and species could explain more or equal proportions of the variance in the multifunction than the single trait combinations. These results thus indicated that even if there was functional redundancy, some key species might be vital for the functioning.

Instead of finding a relationship between the amount of smoothing and number of species (redundancy) contributing to a modality or trait combination, as was hypothesized (1), we observed a reliance on the dominance patterns of the species included in each trait combination or modality. For example, the trait combination mixing*L displayed less patchiness across the landscape than the trait combination *surface modification* (Figures 2 and 3), despite higher number of species within *surface modification*. This was likely due to some overlap between the two modalities within mixing*L and that they had many species in common of the few species in total. The two most abundant species, *Austrovenus stutchburyi* and *Macomona liliana,* drove the spatial patterns of the modalities, and thus, also the spatial pattern of the trait combination mixing*L. Similar indications could also be seen in some modalities included in the trait combination *surface modification*. In two of the modalities (*mound* and *trough*), the smoothed patterns (indicated by large‐patch patterns, Figure 3 and Appendix S3) were driven by dominant species (*Orbinia papillosa* and *M. liliana*, respectively), whereas in two other modalities (*permanent burrow* and *tube structure*), all species expressed a high patchiness and many species were included and/or the species had more even abundances, which then resulted in patchy spatial patterns in the modalities (Figure 3 and Appendix S3). Thus, the results indicated that if a modality includes an abundant species with a high contribution to the modality and which is distributed in large patches, this species will drive the distribution of the modality and mask the patchiness of the other less abundant species (i.e., smoothed distribution). Whereas, if a modality includes many species with smaller patch patterns and even abundances, the modality will also show a more heterogeneous distribution across the seafloor.

The ability of a few species and individual modalities to explain more of the variability in our multifunction than the trait combinations (Table 4) was most likely due to the favorable combination of traits some species express for the explored multifunction (particularly *Austrovenus stutchburyi* in this case). Thus, unlike hypothesized (2), the ability to predict the ecosystem functioning was actually smaller when using the larger‐scale, more homogeneous patterns provided by modalities and trait combinations, and stronger relationships were found when using the presence of the individual dominant species. The use of trait combinations or functional groups has a relatively long tradition within the field of ecology (e.g., Fauchald & Jumars, 1979; Wilson, 1999), but the concept and the methods to form these groups are still being developed (Butterfield & Suding, 2013; Murray, Douglas, & Solan, 2014; Törnroos & Bonsdorff, 2012). Earlier studies often based the classification on trophic groups (e.g., Cardinale et al., 2006; Hairston, Smith, & Slobodkin, 1960; Hunt, 1925), whereas today, the classifications are often based on a variety of functional traits that addresses specific research questions (e.g., Harris, Pilditch, Greenfield, Moon, & Kröncke, 2016; Thrush et al., 2017). Functional groups are suggested to be especially useful when describing potential functionality and resilience across larger scales and environmental gradients (e.g., Greenfield et al., 2016; Villnäs, Hewitt, Snickars, Westerbom, & Norkko, 2018). However, in this study, the trait combinations did not work well overall (i.e., low explanation of the multifunction), which might be more common than generally expected. A similar result was reported by Norkko et al. (2015) regarding the Bioturbation potential index (cf. Solan et al., 2004), where the index was shown to not capture the variance within solute fluxes across a gradient of increasing hypoxia. Instead individual species were best at explaining nutrient dynamics both under hypoxic and oxic conditions.

Functional groups are often used to infer ecosystem functioning in order to overcome logistical difficulties of directly measuring ecosystem functions on larger scales. Quantitative links between specific traits and ecosystem functions are, however, not always that well established (Murray et al., 2014; Snelgrove et al., 2014), and species appearing to perform similar functions (i.e., be redundant) might play different roles depending on the habitat or changing environmental conditions (Needham, Pilditch, Lohrer, & Thrush, 2011; Vaughn, Spooner, & Galbraith, 2007; Walker, 1992; Wellnitz & Poff, 2001). A recent study by Thrush et al. (2017) investigated how biodiversity–ecosystem function relationships were affected by changing nutrient regimes within sediments. Their results indicated individual traits, functional diversity, species richness, and key species all play roles in defining ecosystem function relationships associated with sediment nitrogen processing. Importantly, the relative contribution of the different biodiversity measures changed with altered nutrient regimes. These results thus suggest that a variety of biodiversity descriptors are needed to obtain a holistic picture of BEF relationships, but also that it would be important to assess the context‐dependence of the links between function and specific biodiversity descriptors. It is further important to address several different ecosystem functions (such as primary and secondary production, decomposition, and nutrient recycling), as the BEF relationships are likely to be dependent on the function in question.

Current evidence suggests that even if there is functional redundancy among functionally similar species, the identity of some species may play a pivotal role for the functioning of our ecosystems (e.g., Sandwell, Pilditch, & Lohrer, 2009; Smith & Knapp, 2003). In this study, especially *Austrovenus stutchburyi* and also *Macomona liliana* are typically among the most abundant and biomass dominant species living in New Zealand sandflats, and they have been shown in several studies to have an effect on their surrounding environment and ecosystem functioning (Jones, Pilditch, Bruesewitz, & Lohrer, 2011; Pratt, Pilditch, Lohrer, Thrush, & Kraan, 2015; Sandwell et al., 2009; Thrush et al., 2006). Importantly, *M. liliana* although abundant was not a significant predictor for this particular combination of solute fluxes. The high abundance of this shellfish is driven by high juvenile density, skewing the importance of abundance relative to size and adult living position in the sediment. High abundance of a species does not automatically translate into a large influence on ecosystem functioning. Many studies have, however, demonstrated significant identity effects on functioning (Stachowicz, Bruno, & Duffy, 2007), with the composition of species equally or even more important than species richness (Bruno, Boyer, Duffy, Lee, & Kertesz, 2005; Gammal et al., 2019; Gammal, Norkko, Pilditch, & Norkko, 2017; Gustafsson & Boström, 2009).

Real‐world ecosystems, however, depend on multiple and simultaneously operating functions, which suggests that several complementary species are needed to underpin these functions (e.g., Gamfeldt, Hillebrand, & Jonsson, 2008; Hector & Bagchi, 2007). Different functions, even if closely related, similar to the functions included in the multifunction of this study, have been indicated to be dependent on different aspects of biodiversity (Murray et al., 2014). Functional redundancy when multiple functions are considered on larger and longer scales is thus likely much lower than the redundancy for single functions (Gamfeldt et al., 2008; Rosenfeld, 2002), highlighting that ongoing biodiversity loss could have far graver consequences for the overall functioning of ecosystems than earlier presumed (Reiss et al., 2009).

## CONFLICT OF INTEREST

The authors have declared that no conflict of interest exists.

## AUTHOR CONTRIBUTION

**Johanna Gammal:** Conceptualization (equal); Formal analysis (equal); Investigation (equal); Writing‐original draft (lead). **Judi Hewitt:** Conceptualization (equal); Data curation (equal); Formal analysis (equal); Investigation (equal); Methodology (equal); Writing‐review & editing (equal). **Joanna Norkko:** Conceptualization (equal); Investigation (equal); Writing‐review & editing (equal). **Alf Norkko:** Conceptualization (equal); Investigation (equal); Writing‐review & editing (equal). **Simon Thrush:** Conceptualization (equal); Data curation (equal); Investigation (equal); Methodology (equal); Writing‐review & editing (equal).

## Supporting information

Appendix S1‐S5

## Data Availability

Data are available from Dryad Digital Repository, https://doi.org/10.5061/dryad.05d0q. Thrush et al. (2017): Changes in the location of biodiversity–ecosystem function hot spots across the seafloor landscape with increasing sediment nutrient loading. And from PANGAEA, https://doi.org/10.1594/PANGAEA.903448. Kraan et al. (2015): Multi‐scale data on intertidal macrobenthic biodiversity and environmental features in Kaipara, Tauranga, and Manukau Harbours, New Zealand.
