# Supplementary material for: Does the use of biological traits predict a smooth landscape of ecosystem functioning?
Source: Ecol Evol. 2020 Aug 20;10(19):10395–407. doi: 10.1002/ece3.6696 (PMC7548162; doi:10.1002/ece3.6696)
Supplement: Supplementary file 1 — Appendix S1‐S5 [file ECE3-10-10395-s001.pdf]

SUPPLEMENTARY MATERIAL

TITLE: Does the use of biological traits predict a smooth landscape of ecosystem functioning?

AUTHORS: Johanna Gammal, Judi Hewitt, Joanna Norkko, Alf Norkko, Simon Thrush

Appendix 1. Table of all trait combinations that were tested and the included modalities.

Trait combination	Modality
Mixing	
	Surface to depth
	Depth to surface
Mixing*L	
	Surface to depth by large taxa (size >20 mm)
	Depth to surface by large taxa (size >20 mm)
Mixing*size*motility	
	Surface to depth weighted by size class and motility mode
	Depth to surface weighted by size class and motility mode
	Small (<5 mm) = value 1
	Medium (5–20 mm) = value 2
	Large (>20 mm) = value 3
	Sedentary/movement in a fixed tube = value 1
	Limited movement, usually in sediment = value 2
	Freely motile on or in sediment = value 3
Surface modification	
	Permanent burrow
	Tube structure
	Simple hole or pit
	Mound
	Trough

Appendix 2. Species list of the included taxa, the summed abundance and frequency of occurrence across the whole sandflat (400 samples), and the trait combination each species were referred to. For specific modality allocations see Thrush et al. 2017 *Proc Royal Soc. B*

Species	Phylum/Class	Summed abundance	Occurrence	Sediment mixing			Surface modification
				Mixing	Mixing*L	Mixing*size*mobility	
Aglaophamus macroura	Polychaeta	207	137	x		x	
Alpheus sp.	Eucarida, Decapoda	1	1	x		x	x
Amalda australis	Gastropoda	1	1	x	x	x	x
Aonides trifida	Polychaeta	3915	229	x		x	
Aricidea sp.	Polychaeta	73	40	x		x	x
Armandia maculata	Polychaeta	30	13	x		x	x
Arthritica bifurca	Bivalvia	70	28				x
Asychis sp.	Polychaeta	9	8	x	x	x	x
Austrohelice crassa	Eucarida, Decapoda	8	8	x	x	x	x
Austrovenus stutchburyi	Bivalvia	929	218	x	x	x	x
Barantolla lepte	Polychaeta	4	4	x		x	x
Boccardia syrtis	Polychaeta	472	56	x		x	x
Bumpy cirrisyllid	Polychaeta	454	135	x		x	
Callianassa sp.	Eucarida, Decapoda	3	2				x
Capitella sp.	Polychaeta	34	28	x		x	x
Ceratonereis sp.	Polychaeta	126	59	x		x	x
Chiton glaucus	Polyplacophora	6	5				x
Cirratulidae	Polychaeta	7	6	x		x	x
Colurostylis lemurum	Peracarida	556	220				x
Cominella adspersa	Gastropoda	3	3				x
Cominella glandiformis	Gastropoda	94	69				x
Corophium sp.	Peracarida	20	5	x		x	x
Cossura consimilis	Polychaeta	5	2				x
Cyclapsis thomsoni	Peracarida	30	15				x
Cyclomactra ovata	Bivalvia	34	26	x	x	x	x
Diastylopsis elongata	Peracarida	0	0				x
Diloma subrostrata	Gastropoda	19	17				x
Eatoniella sp.	Gastropoda	13	11				x
Edwardsia sp.	Cnidaria	3	3				x
Euchone sp.	Polychaeta	771	105				x
Eunicidae	Polychaeta	0	0	x		x	
Euterebra tristis	Gastropoda	13	12				x
Felaniella zelandica	Bivalvia	2	2				x
flat worm	Platyhelminthes	0	0				x

Gammaropsis sp.	Peracarida	1	1	x		x	x
Glycera americana	Polychaeta	18	18	x	x	x	
Glycinde grahami	Polychaeta	1	1	x		x	
Glycinde trifida	Polychaeta	1	1	x		x	
Haminoea zelandica	Gastropoda	6	6				x
Harmothoe sp.	Polychaeta	0	0	x		x	
Hemigrapsus crenulatus	Eucarida, Decapoda	0	0	x	x	x	x
Hemigrapsus edwardsi	Eucarida, Decapoda	1	1				x
Hemiplax hirtipes	Eucarida, Decapoda	41	33	x	x	x	x
Hesionidae	Polychaeta	222	115	x		x	
Heteromastus filiformis	Polychaeta	575	218	x		x	x
Lasaea parangaensis	Bivalvia	273	55				x
Lepidastheniella comma	Polychaeta	0	0				x
Lepidonotinae	Polychaeta	3	3	x		x	
Levinsinia gracilis	Polychaeta	4	3	x		x	
Limnoperna pulex	Bivalvia	18	4				x
Lumbrineridae	Polychaeta	2	2	x		x	x
Macomona liliana	Bivalvia	1952	379	x	x	x	x
Macroclymenella stewartensis	Polychaeta	373	138	x		x	x
Magelona dakini	Polychaeta	550	196	x		x	
Micrelenchus tenebrosus	Gastropoda	26	17				x
Musculista senhousia	Bivalvia	44	23	x		x	x
Mysid shrimp	Peracarida	77	54				x
Nassarius burchardi	Gastropoda	0	0				x
Nemertean sp.	Nemertea	808	275	x		x	
Neoguraleus sinclairii	Gastropoda	4	4				x
Nicon aestuariensis	Polychaeta	80	48	x		x	x
Notoacmea scapha	Gastropoda	110	70				x
Notomastus sp.	Polychaeta	22	16	x		x	x
Nucula hartvigiana	Bivalvia	680	162				x
Nudibranch sp.	Gastropoda	0	0				x
Oligochaeta FAT	Oligochaeta	1	1	x		x	x
Oligochaeta THIN	Oligochaeta	82	59	x		x	x
Ophiuroida sp.	Ophiuroidea	2	2				x
Orbinia papillosa	Polychaeta	300	116	x		x	x
Owenia petersonae	Polychaeta	490	119				x
Oxydromus angustifrons	Polychaeta	1	1	x		x	
Paphies australis	Bivalvia	1542	171				x
Paracorophium excavatum	Peracarida	18	4	x		x	x
Paradoneis lyra	Polychaeta	18	15	x		x	x

Patiriella regularis	Asteroidea	1	1				x
Pectinaria australis	Polychaeta	5	5				x
Perinereis vallata	Polychaeta	20	17	x		x	x
Phoronis sp.	Phoronida	415	121				x
Phoxocephalidae	Peracarida	34	17	x		x	
Phyllodocidae	Polychaeta	1	1	x		x	
Platynereis australis	Polychaeta	74	51	x	x	x	x
Polydora sp.	Polychaeta	0	0	x		x	x
Prionospio aucklandica	Polychaeta	566	124	x		x	
Pseudopolydora FAT	Polychaeta	169	75	x		x	
Pseudopolydora THIN	Polychaeta	416	30	x		x	x
Pyromaia sp	Eucarida, Decapoda	0	0	x		x	
Scolecolepides benhami	Polychaeta	90	65	x		x	x
Scolelepis sp.	Polychaeta	17	12	x		x	
Scoloplos cylindrifer	Polychaeta	2	2	x		x	x
Soletellina siliqua	Bivalvia	1157	197				x
Sphaerosyllis semiverrucosa	Polychaeta	0	0	x		x	
Squilla armata	Eucarida, Decapoda	2	2	x		x	x
Syllidae	Polychaeta	0	0	x		x	
Theora lubrica	Bivalvia	4	3	x		x	x
Torridoharpinia hurleyi	Peracarida	57	45	x		x	
Travisia olens	Polychaeta	94	70				x
Trochodota dendyi	Echinoidea	223	129	x		x	x
Turbonilla aoteana	Gastropoda	3	3				x
Xymene plebeius	Gastropoda	6	6				x
Zeacumantus lutulentus	Gastropoda	152	93				x

Appendix 3. The interpreted spatial patterns of the trait combinations, modalities and species based on the Moran's I correlograms (original correlograms in Appendix 4), and the contribution (%) of each species to the modalities, based on trait weighted abundances, across the whole sandflat.

Variable	Spatial patch pattern	Contribution (%)
Mixing*L	300 m patches	
Surface to depth	50 m in 300 m	
Austrovenus stutchburyi	50–70 in 300 m	54.6
Macomona liliana	300 m	34.8
Platynereis australis	70–90 m on gradient	3.3
Glycera americana	heterogeneous patches 50–100 m	0.8
Cyclomactra ovata	10–50 m on gradient	6.1
Others	30 m in 100 m	0.5
Depth to surface	300 m	
Austrovenus stutchburyi	50–70 m in 300 m	20.0
Macomona liliana	300 m	76.4
Hemiplax hirtipes	30–50 m	1.3
Platynereis australis	70–90 m on gradient	1.2
Glycera americana	heterogeneous patches 50–100 m	0.3
Others	30 m in 100 m	0.8
Surface modification	30–90 in 300–400 m	
Permanent burrow	30–90 m	
Aricidea sp.	10–50 poss 170 m	5.2
Capitella sp.	10–50 m	2.4
Ceratonereis sp.	110–140 on gradient	9.0
Oligochaeta THIN	no pattern	5.9
Nicon aestuariensis	30–90 on gradient	5.7
Heteromastus filiformis	90–100 m on gradient	41.2
Hemiplax hirtipes	30–50 m	1.5
Trochodota dendyi	30–50 in 200–300 m	16.0
Perinereis vallata	no pattern	1.4
Musculista senhousia	70–90 on gradient	1.6
Paradoneis lyra	30–50 m	1.3
Armandia maculata	no pattern	2.2
Notomastus sp.	70–90	1.6
Corophium sp.	no pattern	1.4
Paracorophium excavatum	10 m	1.3
Others	10–50 m	2.2
Simple hole or pit	300–350 m	
Austrovenus stutchburyi	50–70 in 300 m	9.4
Colurostylis lemurum	180–200	11.3
Lasaea parangaensis	50–70 on gradient	5.5
Paphies australis	gradient	31.3
Soletellina siliqua	30–50 in 250 m	23.5
Arthritica bifurca	30–50 in 300 m	1.4
Nucula hartvigiana	70 in 180 in 300 in 400	13.8
Mysid shrimp	10–110 m	1.6
Limnoperna pulex	no pattern	0.4
Cyclomactra ovata	10–50 on gradient	0.7
Cyclaspis thomsoni	10–30 on gradient	0.6
Others	10–50 m	0.4
Tube structure	30–50 on gradient	

Euchone sp.	30–50 on gradient	25.5
Boccardia syrtis	10 m in 110 m	15.6
Owenia petersonae	10–50 on gradient	16.2
Macroclymenella stewartensis	160–180 m	12.3
Platynereis australis	70–90 on gradient	2.4
Phoronis sp.	gradient	13.7
Pseudopolydora THIN	10–70 m	13.8
Others	10–50 m	0.5
Mound	200 m	
Scolecolepides benhami	gradient	20.5
Orbinia papillosa	200–220 m	68.5
Hemiplax hirtipes	30–50 m	4.7
Musculista senhousia	70–90 on gradient	5.0
Others	10–50 m	1.3
Trough	300 m	
Austrovenus stutchburyi	30–90 in 300 m	15.7
Macomona liliana	300 m	65.9
Notoacmea scapha	30–50 m	3.7
Travisia olens	30–50 in 250 m	3.2
Zeacumantus lutulentus	gradient	5.1
Diloma subrostrata	no pattern	0.6
Micrelenchus tenebrosus	50–100 m	0.9
Cominella glandiformis	no pattern	3.2
Eatoniella sp.	no pattern	0.4
Euterebra tristis	10–90 m	0.4
Others	10–50 m	0.8

Appendix 4. Moran’s I correlograms for all species included in the trait combinations *mixing*L* and *surface modification*. Filled symbols indicate significant Moran’s I values.

Species in mixing*L

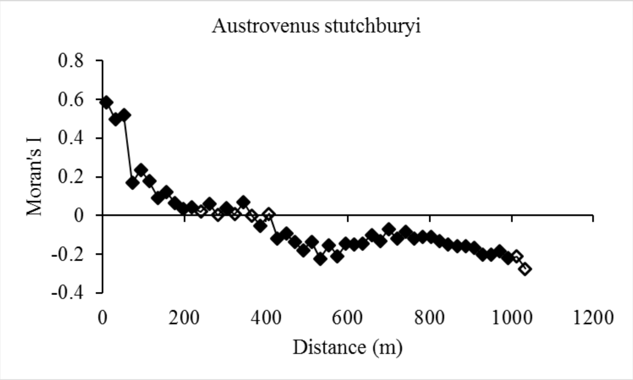

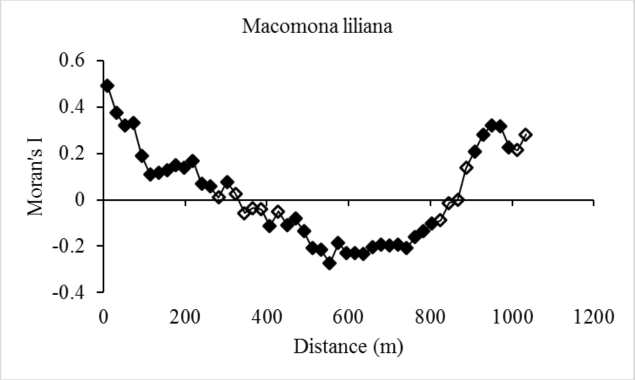

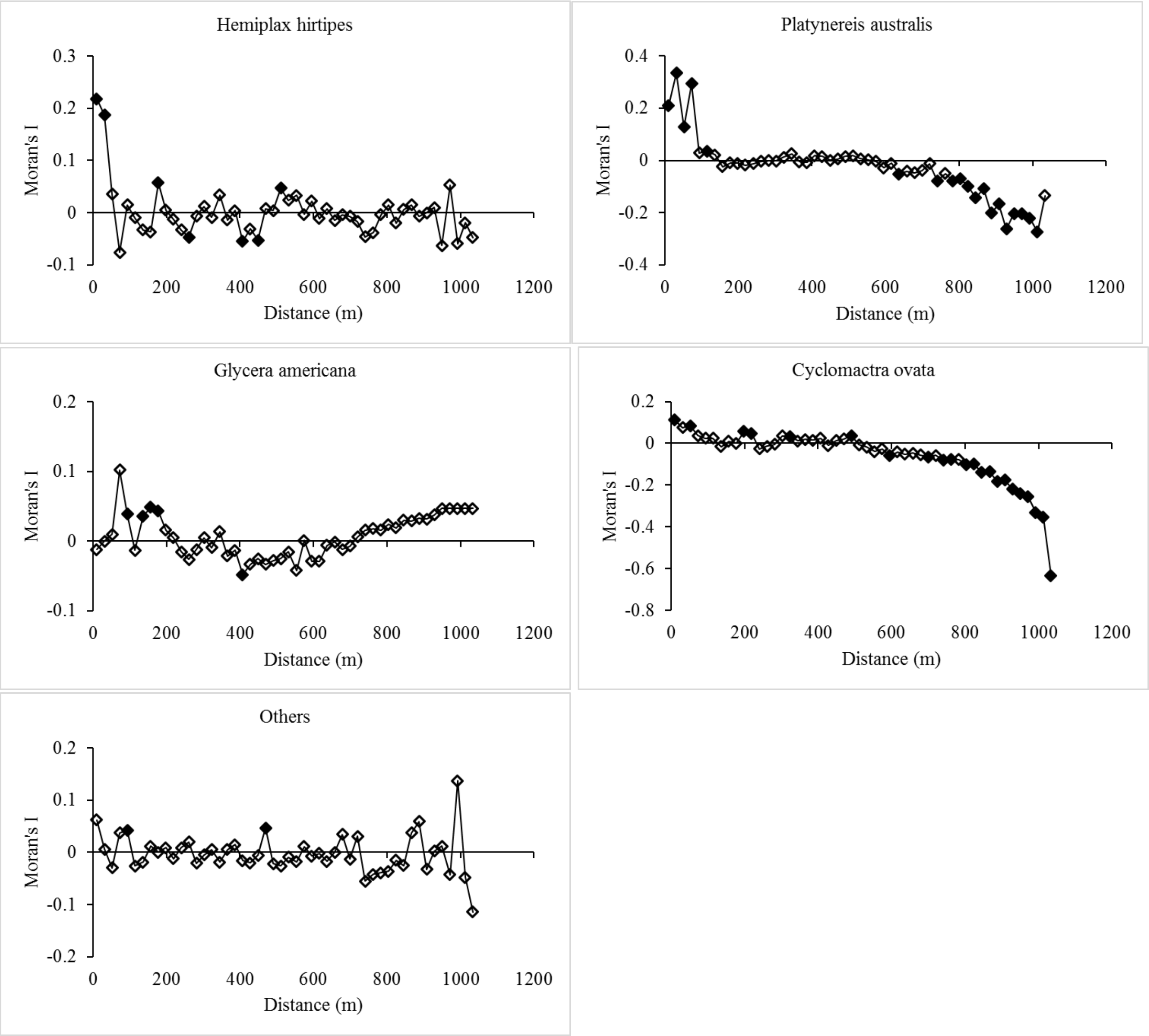
Hemiplax hirtipes
Moran's I
0.3
0.2
0.1
0
-0.1
0
200
400
600
800
1000
1200
Distance (m)
Platynereis australis
Moran's I
0.4
0.2
0
-0.2
-0.4
0
200
400
600
800
1000
1200
Distance (m)
Glycera americana
Moran's I
0.2
0.1
0
-0.1
0
200
400
600
800
1000
1200
Distance (m)
Cyclomactra ovata
Moran's I
0.2
0
-0.2
-0.4
-0.6
-0.8
0
200
400
600
800
1000
1200
Distance (m)
Others
Moran's I
0.2
0.1
0
-0.1
-0.2
0
200
400
600
800
1000
1200
Distance (m)

Species in surface modification

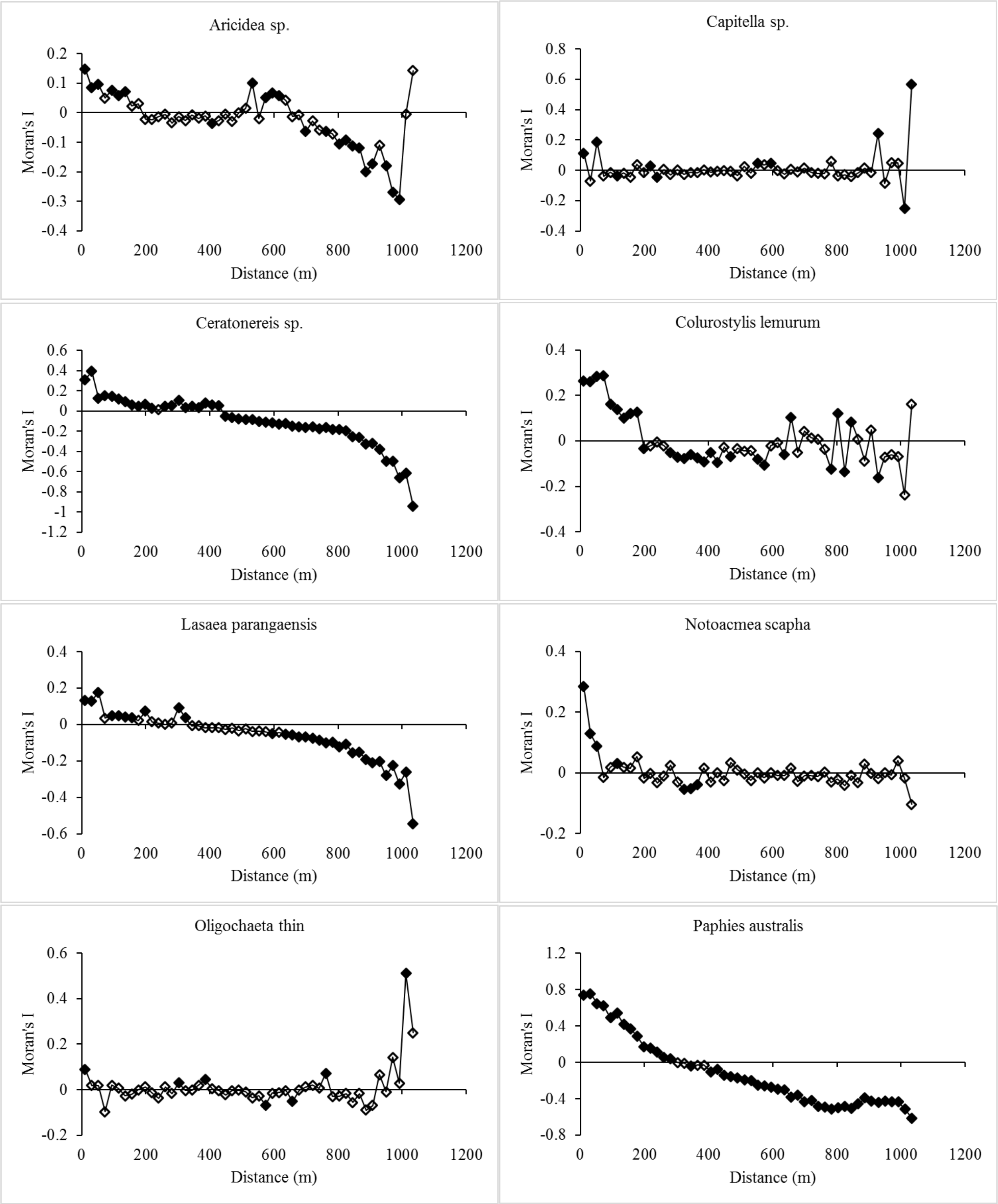

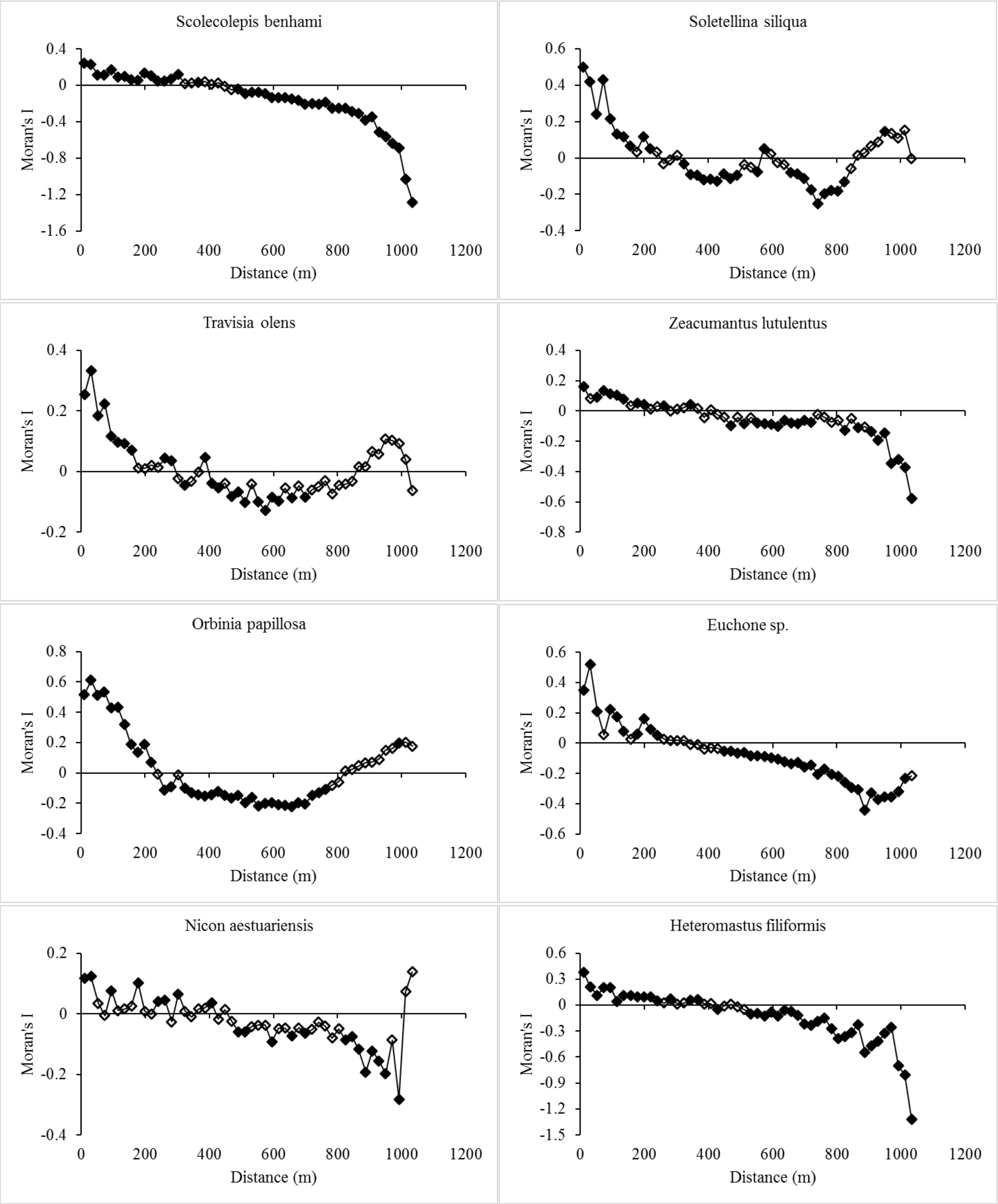

Scolecolepis benhami
Soletellina siliqua
Travisia olens
Zeacumantus lutulentus
Orbinia papillosa
Euchone sp.
Nicon aestuariensis
Heteromastus filiformis
Moran's I
Distance (m)
0
200
400
600
800
1000
1200
0.4
0
-0.4
-0.8
-1.2
-1.6
0.6
0.2
-0.2
-0.6
-0.8
0.8
0.3
-0.3
-0.9
-1.5

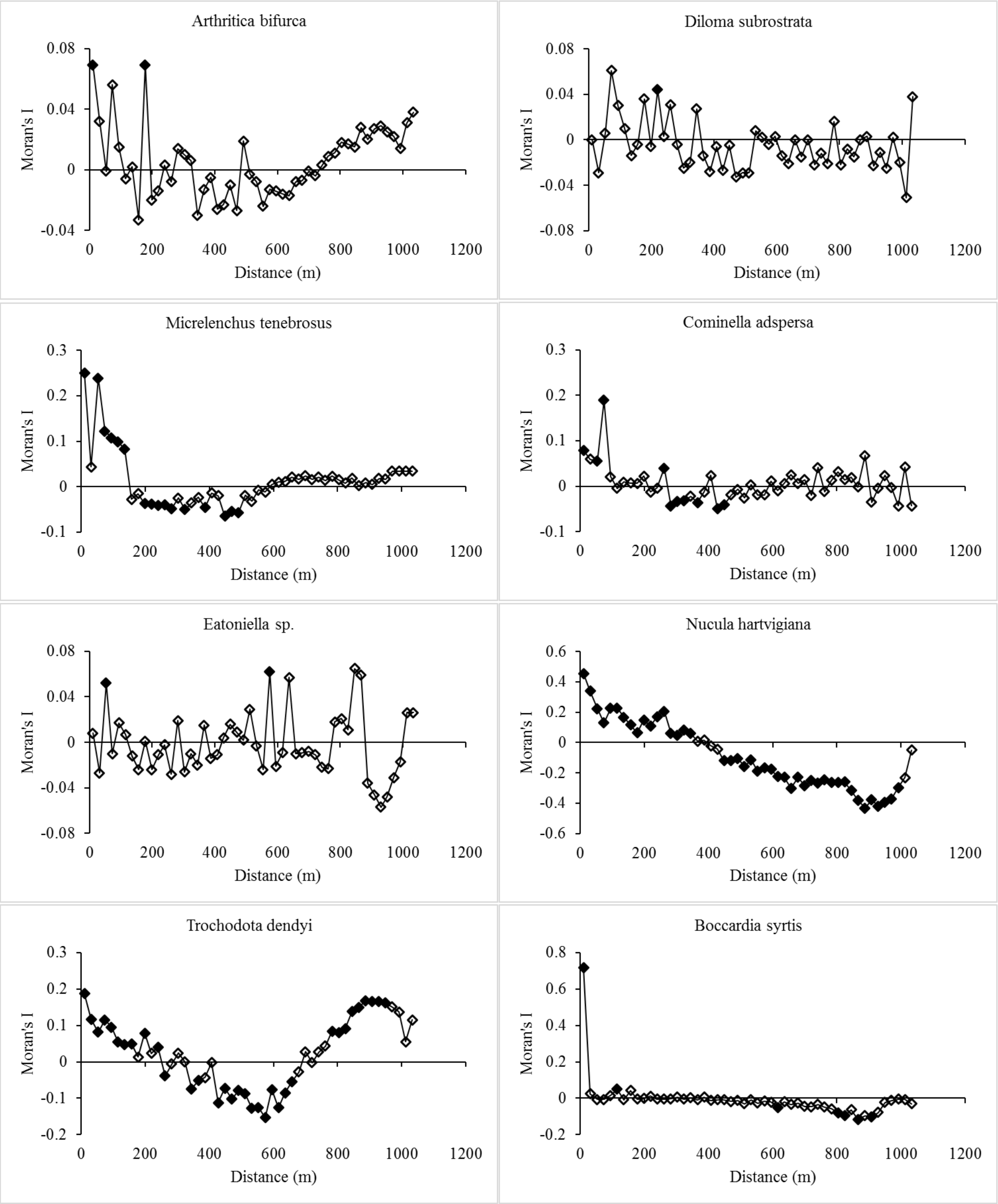

Arthritica bifurca
Diloma subrostrata
Micrelenchus tenebrosus
Cominella adspersa
Eatoniella sp.
Nucula hartvigiana
Trochodota dendyi
Boccardia syrtis
Moran's I
Distance (m)
0
200
400
600
800
1000
1200
0.08
0.04
-0.04
-0.08
0.3
0.2
0.1
-0.1
-0.2
0.6
0.4
-0.4
-0.6
0.8

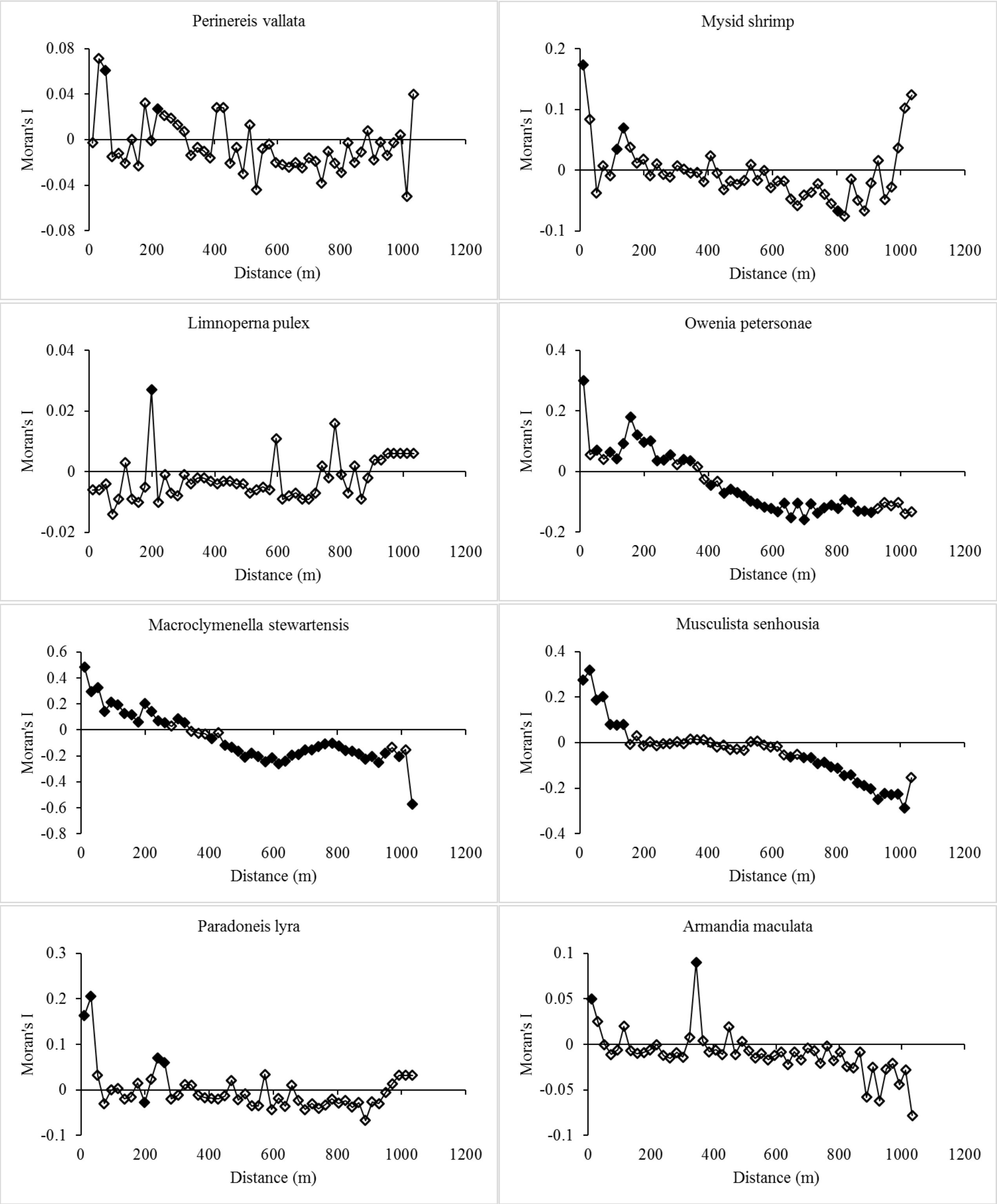
Perinereis vallata
Moran's I
0.08
0.04
0
-0.04
-0.08
0
200
400
600
800
1000
1200
Distance (m)
Mysid shrimp
Moran's I
0.2
0.1
0
-0.1
0
200
400
600
800
1000
1200
Distance (m)
Limnoperna pulex
Moran's I
0.04
0.02
0
-0.02
0
200
400
600
800
1000
1200
Distance (m)
Owenia petersonae
Moran's I
0.4
0.2
0
-0.2
0
200
400
600
800
1000
1200
Distance (m)
Macroclymenella stewartensis
Moran's I
0.6
0.4
0.2
0
-0.2
-0.4
-0.6
-0.8
0
200
400
600
800
1000
1200
Distance (m)
Musculista senhousia
Moran's I
0.4
0.2
0
-0.2
-0.4
0
200
400
600
800
1000
1200
Distance (m)
Paradoneis lyra
Moran's I
0.3
0.2
0.1
0
-0.1
0
200
400
600
800
1000
1200
Distance (m)
Armandia maculata
Moran's I
0.1
0.05
0
-0.05
-0.1
0
200
400
600
800
1000
1200
Distance (m)

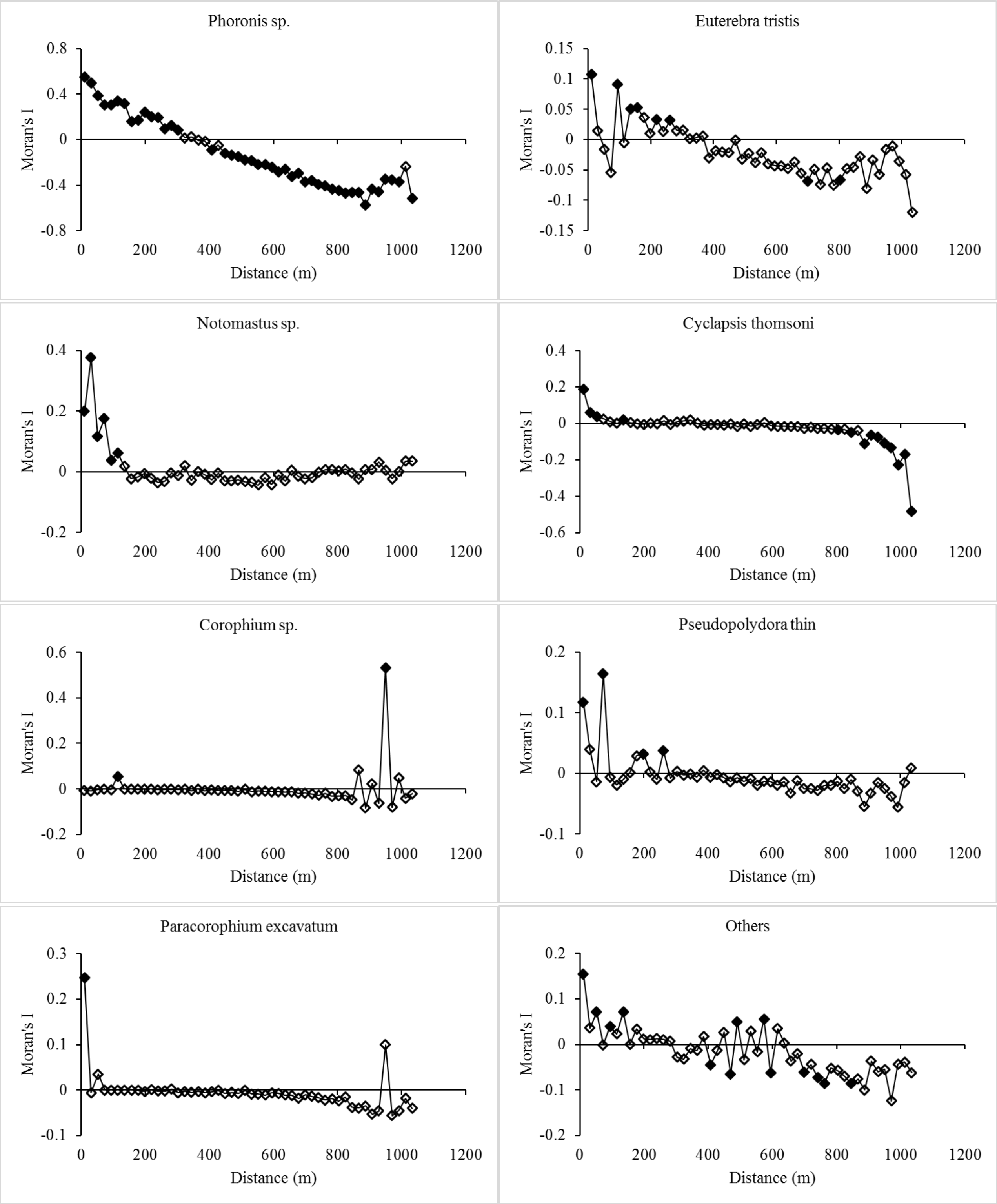
Phoronis sp.
Euterebra tristis
Notomastus sp.
Cyclapsis thomsoni
Corophium sp.
Pseudopolydora thin
Paracorophium excavatum
Others
Moran's I
Distance (m)
0
200
400
600
800
1000
1200

Appendix 5. Spearman correlations between the trait combination surface modification and the included modalities and species. Correlations that were >|0.1| are reported, and Spearman's rho ≥0.5 are in bold.

	Surface modification	Permanent burrow	Tube structure	Aricidea sp.	Austrovenus stutchburyi	Capitella sp.	Ceratonereis sp.	Colurostylis lemurum	Lasaea parangaensis	Macomona liliana	Notoacmea scapha	Oligochaeta thin
Aricidea sp.		0.14										
Capitella sp.				0.11								
Lasaea parangaensis					0.14		0.29					
Macomona liliana	0.12											
Notoacmea scapha					0.11							
Oligochaeta thin		0.15		0.14								
Paphies australis				-0.11	0.13		0.24	0.11	0.35			
Scolecolepides benhami							0.28		0.30			
Soletellina siliqua				-0.10	0.15				0.12	0.12		
Travisia olens					0.10				0.12			
Zeacumantus lutulentus					0.11	0.10						
Orbinia papillosa							0.12					
Euchone sp.	0.10		**0.56**	0.24			-0.13		-0.12			0.13
Nicon aestuariensis							0.11		0.15			0.24
Heteromastus filiformis		0.39		0.16								0.13
Arthritica bifurca						0.17						
Diloma subrostrata					0.11							
Micrelenchus tenebrosus					0.17						0.12	
Cominella glandiformis											0.13	
Eatoniella sp.											0.17	
Hemiplax hirtipes											0.13	
Nucula hartvigiana				0.14			-0.13		-0.13			
Trochodota dendyi												
Boccardia syrtis			0.33	0.11			-0.12		-0.13			
Perinereis vallata						0.14						
Limnoperna pulex												
Owenia petersonae	0.11		**0.53**	0.10			-0.15		-0.17			
Macroclymenella stewartensis			**0.55**	0.10			-0.14		-0.15			
Musculista senhousia	0.12											
Platynereis australis							-0.12		-0.11		0.11	
Paradoneis lyra												
Armandia maculata	0.11											0.10
Cyclomactra ovata				0.10								
Phoronis sp.	0.10		**0.55**				-0.15		-0.16			
Euterebra tristis												
Notomastus sp.												
Cyclaspis thomsoni	0.10			0.24								0.11
Corophium sp.		0.10		0.11								
Pseudopolydora thin			0.25				-0.10					
Paracorophium excavatum							0.12		0.12			
Surf others <10 ind						0.11						

	Paphies australis	Scolecolepides benhami	Soletellina siliqua	Travisia olens	Zeacumantus lutulentus	Orbinia papillosa	Euchone sp.	Nicon aestuariensis	Heteromastus filiformis	Arthritica bifurca	Diloma subrostrata	Micrelenchus tenebrosus
Scolecolepides benhami	0.24											
Soletellina siliqua	0.28											
Travisia olens			0.24									
Zeacumantus lutulentus	0.11			0.15								
Orbinia papillosa	0.31	0.10	0.36	0.17	0.13							
Euchone sp.	-0.16	-0.11	-0.11	-0.11	-0.14	-0.14						
Nicon aestuariensis	0.10	0.11										
Heteromastus filiformis							0.24					
Arthritica bifurca												
Diloma subrostrata										0.12		
Micrelenchus tenebrosus										0.26		
Cominella glandiformis												0.16
Eatoniella sp.												0.11
Hemiplax hirtipes										0.14	0.14	0.19
Nucula hartvigiana		-0.11				-0.11	0.43		0.13			
Trochodota dendyi				0.15	0.12							
Boccardia syrtis	-0.14						0.34		0.14			
Perinereis vallata	0.14									0.13		
Limnoperna pulex												
Owenia petersonae	-0.16	-0.13	-0.11			-0.14	0.41		0.15			
Macroclymenella stewartensis	-0.11	-0.13				-0.11	0.29		0.13			
Musculista senhousia	-0.11						0.26			0.13		
Platynereis australis	-0.11					-0.10	0.14					0.15
Paradoneis lyra							0.20					
Armandia maculata							0.13					
Cyclomactra ovata	-0.10						0.20					
Phoronis sp.	-0.14	-0.13		-0.13	-0.13	-0.14	0.43	-0.10				
Euterebra tristis							0.14					
Notomastus sp.							0.11					
Cyclaspis thomsoni							0.28		0.13			
Corophium sp.		0.12					0.11					
Pseudopolydora thin	-0.11					-0.10	0.25					
Paracorophium excavatum												
Surf others <10 ind	-0.11						0.14			0.14		

	Cominella glandiformis	Eatoniella sp.	Hemiplax hirtipes	Nucula hartvigiana	Trochodota dendyi	Boccardia syrtis	Mysid shrimp	Owenia petersonae	Macroclymenella stewartensis	Musculista senhousia	Platynereis australis	Paradoneis lyra
Hemiplax hirtipes		0.18										
Nucula hartvigiana			0.14									
Trochodota dendyi												
Boccardia syrtis				0.18								
Perinereis vallata												
Limnoperna pulex	0.15											
Owenia petersonae			0.19	0.43		0.35						
Macroclymenella stewartensis				0.29		0.22		0.36				
Musculista senhousia			0.12	0.17		0.25		0.20	0.14			
Platynereis australis	0.13		0.26	0.14		0.11		0.19	0.13	0.27		
Paradoneis lyra				0.14					0.17			
Armandia maculata			0.34			0.14		0.18	0.11		0.26	
Cyclomactra ovata						0.19		0.22	0.14			
Phoronis sp.				0.22		0.27	0.12	0.29	0.33	0.15		
Euterebra tristis						0.20						
Notomastus sp.			0.11					0.18	0.16			0.16
Cyclaspis thomsoni				0.14		0.21		0.15	0.14			0.30
Corophium sp.						0.16						
Pseudopolydora thin			0.11	0.16				0.27	0.18	0.11	0.16	0.15
Paracorophium excavatum			0.13									
Surf others <10 ind				0.16						0.13	0.10	0.17

	Armandia maculata	Cyclomactra ovata	Phoronis sp.	Notomastus sp.	Cyclaspis thomsoni	Corophium sp.	Pseudopolydora thin
Cyclomactra ovata	0.12						
Phoronis sp.		0.13					
Euterebra tristis		0.13	0.11				
Notomastus sp.							
Cyclaspis thomsoni	0.11	0.17	0.13	0.16			
Corophium sp.					0.21		
Pseudopolydora thin	0.23	0.14	0.18	0.17	0.14		
Paracorophium excavatum	0.12			0.10	0.12	0.21	
Surf others <10 ind							0.11
